# Biomarkers in Women's Cancers, Gynecology, and Obstetrics

**DOI:** 10.1155/2014/602340

**Published:** 2014-07-10

**Authors:** Peter A. Fasching, Gottfried E. Konecny, Amanda B. Spurdle

**Affiliations:** ^1^ Department of Gynecology and Obstetrics, Comprehensive Cancer Center EMN, University Hospital Erlangen, Friedrich-Alexander University of Erlangen-Nuremberg, 91054 Erlangen, Germany; ^2^ Division of Hematology-Oncology, Department of Medicine, David Geffen School of Medicine, University of California,Los Angeles, Los Angeles, CA 90095, USA; ^3^ Molecular Cancer Epidemiology Laboratory, Genetics and Computational Biology Division, QIMR Berghofer Medical Research Institute, Royal Brisbane Hospital Post Office, Herston, QLD 4029, Australia

More than 10 years after the publication of the reference sequence of the human genome several advances in biomarker discovery in obstetrics and gynecology and women's cancer research have been made. Genome-wide studies aiming at the identification of pathophysiologically relevant novel genes and pathways have discovered multiple previously unknown molecular mechanisms, and this has fostered a new phase of candidate gene and pathway research that is informed by complex genetic, biological, and bioinformatic data. Technological advances have permitted biomarker research on all levels of systems biology (Table 1). In breast and ovarian cancer large consortia have genotyped germ line DNA of more than 60,000 cancer patients and more than 60,000 controls for more than 200,000 single nucleotide polymorphisms [1, 2]. Moreover, several comprehensive reports have been published that decipher breast, ovarian, and endometrial cancer at all levels of gene regulation [3–5]. The ability to study circulating DNA through noninvasive sampling of blood is one of the most exciting and rapidly advancing fields in obstetrics and gynecology and cancer diagnostics. These advances now also enable the analysis of fetal DNA, which is circulating in the blood stream of the mother [6]. These developments have been driven not only by major technological advances, including the isolation of intact cancer cells and the analysis of cancer cell-derived DNA from blood samples, but also by the improvement of the sensitivity of analytic methods. Analysis of free circulating fetal DNA will change the diagnostic approach in prenatal medicine drastically. Moreover, in cancer research a “liquid biopsy” approach which has evolved most prominently in breast cancer will have a significant impact on early detection of cancer and treatment monitoring as well as understanding treatment failure in the near future [7]. Importantly, however, it is not only the discovery of biomarkers that counts, but also the replication and independent validation of results that allow their application in clinical practice. Last but not least, the increasing complexity of biomarker research and their potential clinical utility in the postgenomic era create a need for education of clinicians, clinical researchers, regulatory authorities, and patients alike. The amount of information requires new approaches, how biomarker use is to be understood and to be explained to those involved [8]. We believe that in this special issue we can provide fascinating reviews and original papers on the topic of biomarkers in obstetrics and gynecology and women's cancers in the postgenomic era. It is clear that this field of research will be of increasing importance to obstetrics and gynecology and women's cancers over the next years. We hope that this special issue with a focus on biomarker research in obstetrics and gynecology and women's cancers will provide all participants involved in the field of biomarker research with a comprehensive update on varied recent developments in this exciting field of research.

*Peter A. Fasching* *Gottfried E. Konecny* *Amanda B. Spurdle*

## Figures and Tables

**Table 1:** Different levels of biomarker research and application.

Analysis	Purpose
Methylation	Methylation analysis can reveal disease specific patterns of gene regulation.
Mutation analysis	Mutation analysis of affected tissues has been shown to be helpful in characterization of tumors and rare diseases.
Copy number variation	Copy number variation has been shown to be helpful in explaining tumor subtypes and identifying fetal chromosomal anomalies.
Chromosomal rearrangements	Chromosomal rearrangements are thought to explain new genomic effects seen in some tumors.
Genetic variation	Many diseases have been described to be partially caused by genetic variation. For many gynecologic diseases risk loci with high and low penetrance have been identified.
RNA expression	RNA expression of normal and diseased tissues has long been used to identify biomarkers for disease stage and prediction of therapies.
Proteomics	Modifications of proteins can be meaningful for their function and reveal additional functional information that supplements analysis of gene expression at the RNA level.
miRNA profiles	The number of small regulatory RNA sequences is growing and miRNAs are now understood as regulators for up to several hundreds of genes at once.
